# Supplementary material for: Pretreatment with a Heat-Killed Probiotic Modulates the NLRP3 Inflammasome and Attenuates Colitis-Associated Colorectal Cancer in Mice
Source: Nutrients. 2019 Feb 28;11(3):516. doi: 10.3390/nu11030516 (PMC6471765; doi:10.3390/nu11030516)
Supplement: Supplementary file 1 [file nutrients-11-00516-s001.pdf]

Supplementary information

Pretreatment with a heat-killed probiotic modulates the NLRP3 inflammasome and prevents colitis-associated colorectal cancer in mice

Authors

I-Che Chung[1], Chun-Nan OuYang[1], Sheng-Ning Yuan[1], Hsin-Chung Lin[2,3], Kuo-Yang Huang[4], Pao-Shu Wu[5,6], Chia-Yuan Liu[6,7,8], Kuen-Jou Tsai[9], Lai-Keng Loi[10], Yu-Jen Chen[8,11], An-Ko Chung[12], David M. Ojcius[13,14,15], Yu-Sun Chang[1,12,16], Lih-Chyang Chen[6]

Authors' Affiliations

[1] Molecular Medicine Research Center, Chang Gung University, Taoyuan 333, Taiwan; ycc0311@gmail.com (I.-C.C.); oychunnan@gmail.com (C.-N.O.); ishucab@gmail.com (S.-N.Y.); ysc@mail.cgu.edu.tw (Y.-S.C.)
[2] Graduate Institute of Medical Sciences, National Defense Medical Center, Taipei 114, Taiwan; hsinchunglin@gmail.com
[3] Division of Clinical Pathology, Department of Pathology, Tri-Service General Hospital, Taipei 114, Taiwan
[4] Graduate Institute of Pathology and Parasitology, National Defense Medical Center, Taipei 114, Taiwan; cguhgy6934@gmail.com
[5] Department of Pathology, Mackay Memorial Hospital, New Taipei City 251, Taiwan; pw2136@gmail.com
[6] Department of Medicine, Mackay Medical College, New Taipei City 252, Taiwan; lihchyang@mmc.edu.tw (L.-C.C.)
[7] Division of Gastroenterology, Department of Internal Medicine, MacKay Memorial Hospital, New Taipei City 251, Taiwan; t109@mmc.edu.tw
[8] Department of Medical Research, MacKay Memorial Hospital, New Taipei City 251, Taiwan
[9] Department of Laboratory Medicine, MacKay Memorial Hospital, Taipei 104, Taiwan; benson@mmh.org.tw
[10] Department of Dentistry, School of Dentistry, National Yang-Ming University, Taipei 112, Taiwan; keng1245@gmail.com
[11] Department of Radiation Oncology, Mackay Memorial Hospital, New Taipei City 251, Taiwan; chenmdphd@gmail.com
[12] Graduate Institute of Biomedical Sciences, College of Medicine, Chang Gung University, Taoyuan 333, Taiwan; sevemthday@hotmail.com (A.-K.C.)
[13] Department of Biomedical Sciences, University of the Pacific Arthur A. Dugoni School of Dentistry, San Francisco, CA 94103, USA; dojcius@pacific.edu
[14] Center for Molecular and Clinical Immunology, Chang Gung University, Taoyuan 333, Taiwan
[15] Chang Gung Immunology Consortium, Chang Gung Memorial Hospital, Linkou 333, Taiwan
[16] Department of Otolaryngology-Head & Neck Surgery, Chang Gung Memorial Hospital, Linkou 333, Taiwan

Corresponding author

* Correspondence: lihchyang@mmc.edu.tw; Tel.: 886-2-26360303 ext 1225 (L.-C.C.); ysc@mail.cgu.edu.tw; Tel.: 886-3-211-8800 ext 5131 (Y.-S.C.)

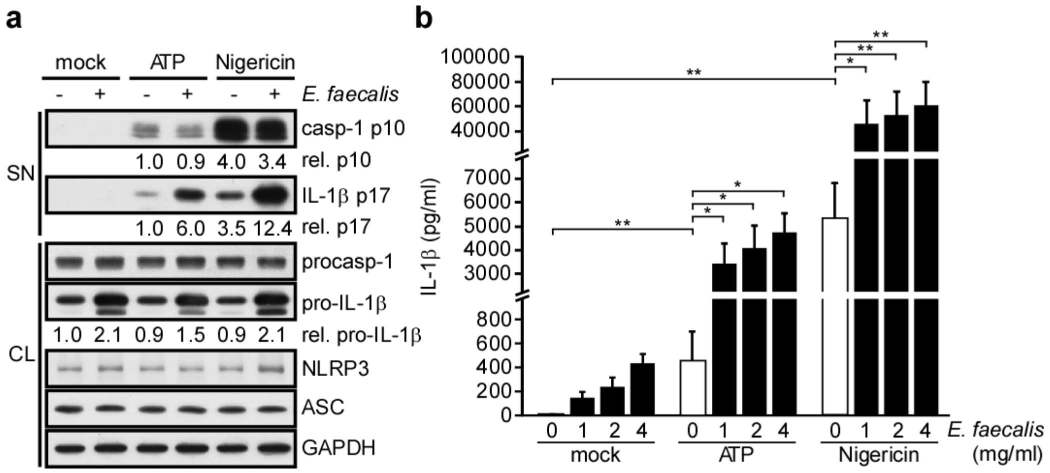

Figure S1. ***E. faecalis*** **enhances ATP- and nigericin-induced IL-1β secretion through upregulation of pro-IL-1β expression**. **(a and b)** THP-1-derived macrophages were pretreated with the indicated amount of *E. faecalis* for 24 h and then stimulated with ATP (for 4 h) or nigericin (for 1 h). **(a)** Immunoblot analysis of NLRP3 inflammasome molecules in cell supernatants (SN) and cell lysates (CL) The western blot is a representative of three independent experiments. Immunoblot images were quantified with the ImageJ software. **(b)** ELISA of IL-1β in the supernatant are shown. Symbols: *, $P < 0.05$; and **, $P < 0.01$. All results are presented as the mean ± SD of three independent experiments and were analyzed with the Student's t test. Abbreviations: procasp-1, p45 precursor of caspase-1; casp-1 p10, active caspase-1 subunits; IL-1β p17, secreted mature IL-1β; and pro-IL-1β, p31 precursor of IL-1β.

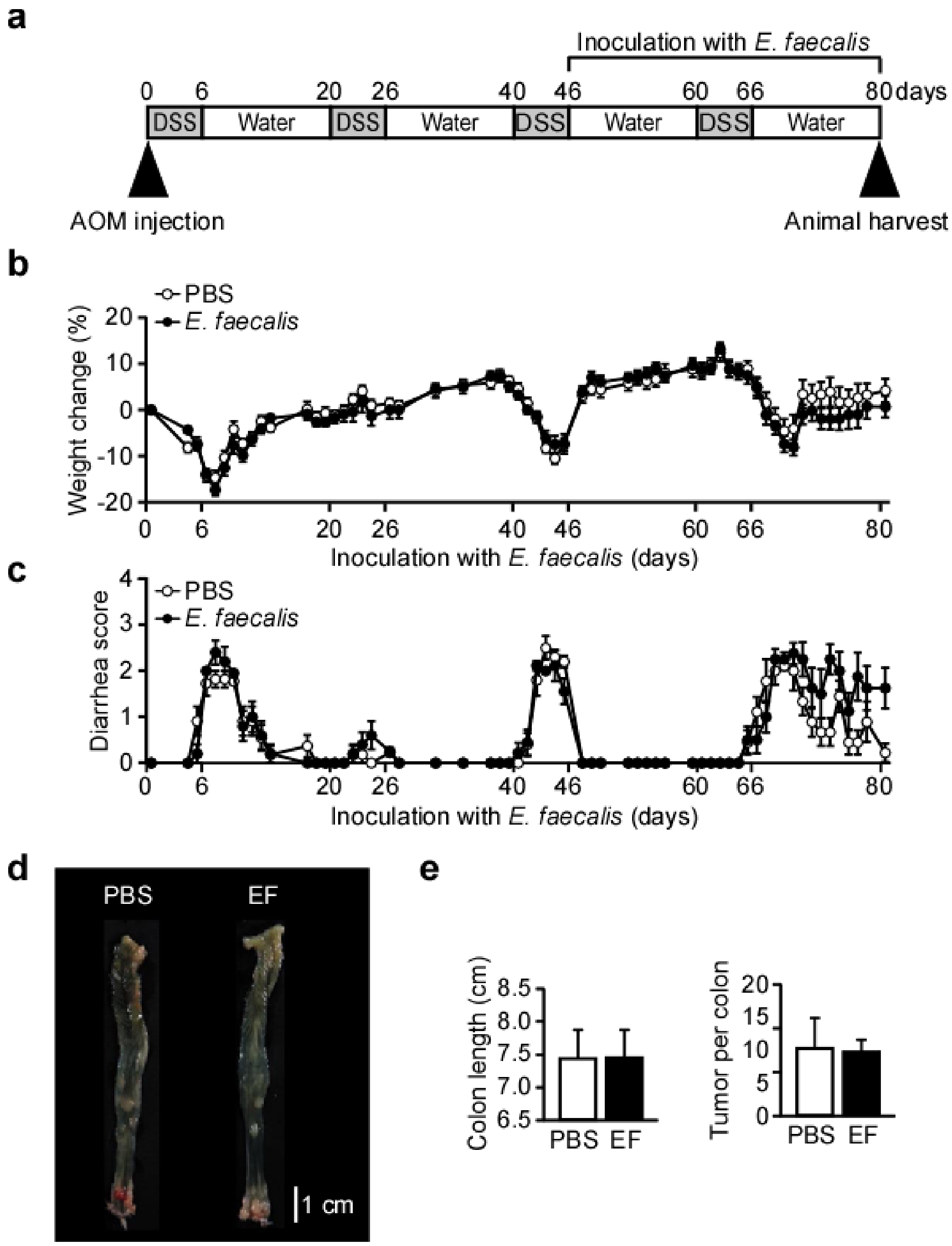

Figure S2. *E. faecalis* treatment does not appear to affect pre-existing colitis-associated CRC. Schematic presentation of the mouse model of colitis-associated CRC. Mice were injected intraperitoneally with AOM prior to beginning the first of four cycles of DSS in the drinking water. One cycle was defined as 6 days of DSS followed by 14 days of water. Mice were orally inoculated with *E. faecalis* every day, starting at the end of the third DSS treatment. Eighty days after the start of DSS treatment, all mice were sacrificed; n=11 for the PBS control group and n=10 for the *E. faecalis*-treated group. **(b and c)** Percent weight change **(b)** and diarrhea scores **(c)** were monitored daily for all mice. **(d)** Representative images of colons. **(e)** Colon length and tumor number. Scale bars, 1 cm. Symbols: *, $P < 0.05$; and **, $P < 0.01$.
